# The Gastrointestinal Tract Is a Major Source of Echinocandin Drug Resistance in a Murine Model of Candida glabrata Colonization and Systemic Dissemination

**DOI:** 10.1128/AAC.01412-17

**Published:** 2017-11-22

**Authors:** Kelley R. Healey, Yoji Nagasaki, Matthew Zimmerman, Milena Kordalewska, Steven Park, Yanan Zhao, David S. Perlin

**Affiliations:** Public Health Research Institute, New Jersey Medical School, Rutgers Biomedical and Health Sciences, Newark, New Jersey, USA

**Keywords:** Candida glabrata, antifungal resistance, echinocandin, intestinal colonization, nikkomycin, systemic dissemination

## Abstract

Candida species are a part of the human microbiome and can cause systemic infection upon immune suppression. Candida glabrata infections are increasing and have greater rates of antifungal resistance than other species. Here, we present a C. glabrata gastrointestinal (GI) colonization model to explore whether colonized yeast exposed to caspofungin, an echinocandin antifungal, develop characteristic resistance mutations and, upon immunosuppression, breakthrough causing systemic infection. Daily therapeutic dosing (5 mg/kg of body weight) of caspofungin resulted in no reduction in fecal burdens, organ breakthrough rates similar to control groups, and resistance rates (0 to 10%) similar to those reported clinically. Treatment with 20 mg/kg caspofungin initially reduced burdens, but a rebound following 5 to 9 days of treatment was accompanied by high levels of resistance (*FKS1*/*FKS2* mutants). Although breakthrough rates decreased in this group, the same *FKS* mutants were recovered from organs. In an attempt to negate drug tolerance that is critical for resistance development, we cotreated mice with daily caspofungin and the chitin synthase inhibitor nikkomycin Z. The largest reduction (3 log) in GI burdens was obtained within 3 to 5 days of 20 mg/kg caspofungin plus nikkomycin treatment. Yet, echinocandin resistance, characterized by a novel Fks1-L630R substitution, was identified following 5 to 7 days of treatment. Therapeutic caspofungin plus nikkomycin treatment left GI burdens unchanged but significantly reduced organ breakthrough rates (20%; *P* < 0.05). Single-dose pharmacokinetics demonstrated low levels of drug penetration into the GI lumen posttreatment with caspofungin. Overall, we show that C. glabrata echinocandin resistance can arise within the GI tract and that resistant mutants can readily disseminate upon immunosuppression.

## INTRODUCTION

While many pathogenic fungi, such as Aspergillus and Cryptococcus spp., are acquired from the environment, Candida is a natural human commensal living in the gastrointestinal (GI) tract. Most infections are endogenous and occur in immunocompromised patients, such as those undergoing solid-organ or hematopoietic stem cell transplantation or specific cancer treatments. Candida species are a leading cause of fungus-associated morbidity and mortality in these patients (1). Therefore, patients considered at high risk for the development of an invasive fungal infection are commonly placed on antifungal therapy with either triazoles (which target ergosterol biosynthesis) or echinocandins (which target cell wall biosynthesis). C. albicans is the most frequently isolated Candida species, but C. glabrata has emerged as the most common cause of invasive infections in specific subsets of patients, such as hematopoietic stem cell transplant recipients, who are commonly placed on prophylactic antifungal regimens (2–4). Additionally, recent reports have described multidrug-resistant (MDR) C. glabrata isolates in the United States (5–7). MDR isolates demonstrate resistance to two or more classes (triazoles/echinocandins/polyenes) of antifungal drugs.

Administration of an echinocandin is now recommended as the first-line treatment of invasive candidiasis (8). Echinocandins (caspofungin, micafungin, and anidulafungin) target the synthesis of beta-1,3-glucan, a polymer required for cell wall synthesis, and resistance arises through mutations that occur within “hot spot” regions of the catalytic subunits (*FKS1*/*FKS2*) of glucan synthase (9). Multiple mechanisms, including gain-of-fitness mutations (10–12), chromosomal rearrangements (13–15), cell wall fortification through chitin production (16, 17), and heteroresistance (18), have been described as factors contributing to the ability of C. glabrata to survive multiple host and drug pressures. Additionally, our lab recently reported that over half of all C. glabrata clinical strains collected from clinics around the world exhibit a partial mutator phenotype as a result of loss-of-function mutations within the DNA mismatch repair gene *MSH2* (19). The high rate of *MSH2* mutations may be related to the unusually high percentages (20 to 30%) of acquired triazole resistance and emerging MDR associated with C. glabrata infections.

The GI tract serves as a primary site of Candida colonization and upon host immunosuppression can be a source for systemic disease (20–22). The GI tract is also proposed to be a main reservoir of antimicrobial resistance and a potential origin of drug-resistant mutants (21, 23). Therefore, resistant isolates recovered from patient blood or other internal organs may originate from the gut, and insufficient drug exposure might play a role. We have developed a murine model of C. glabrata gastrointestinal colonization and systemic breakthrough in order to better understand how antifungal therapy influences (i) yeast burden levels in the GI tract, (ii) the emergence of drug resistance within the GI tract, and (iii) breakthrough causing systemic dissemination following immunosuppression. This study is a proof of principle that antifungal drug exposure in the GI tract can lead to resistance among colonizing organisms.

## RESULTS

### High-dose echinocandin treatment decreases GI burdens and selects for resistance.

**FIG 1:**
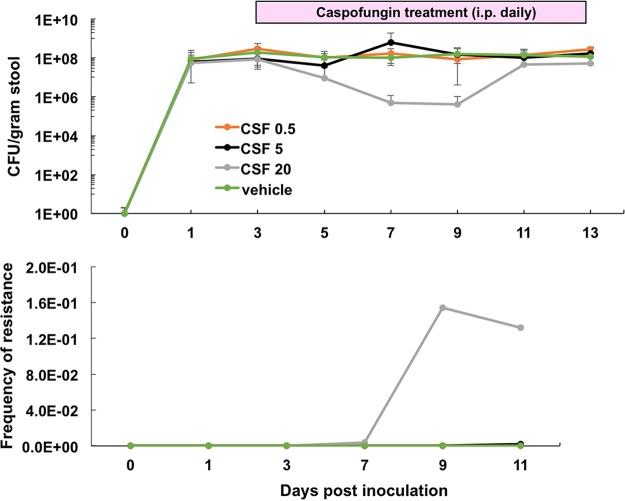
High-dose daily treatments of caspofungin reduce GI burdens but also lead to resistance within 7 days of treatment. Average GI burdens (top) and average frequencies of resistance (bottom) are shown for each treatment group. Ten mice per group were colonized and then treated (i.p.) daily with caspofungin (CSF), as indicated (0.5, 5, or 20 mg/kg). Control vehicle mice were treated i.p. with PBS. Resistance rates were determined by growth on agar plates containing 2 μg/ml caspofungin, and mutations were confirmed through allele-specific molecular beacon analysis (see Fig. S2).

Effective high-burden colonization of mice with C. glabrata required bacterial eradication with daily application of piperacillin-tazobactam (PTZ); removal of the antibiotic decreased the total burden of fungal colonization, as previously demonstrated (19). To assess the effect of echinocandin treatment on GI burden, immunocompetent mice were effectively colonized (10^7^ to 10^8^ CFU/g of stool) with a laboratory-derived mutator strain (Δ*msh2*) on day 0 and then treated intraperitoneally (i.p.) with daily doses of 0.5, 5, or 20 mg/kg of body weight caspofungin beginning on day 3. The vehicle control group was treated with phosphate-buffered saline (PBS) i.p. An approximate 2.5-log decrease in average burden was observed following 5 days of high-dose (20 mg/kg) treatment (Fig. 1, top). However, burdens returned to baseline (∼1 × 10^8^ CFU/g of stool) by day 11. This “rebound” in burden levels was accompanied by the emergence of caspofungin-resistant fecal colonies (Fig. 1, bottom) and the identification of a characteristic echinocandin-resistant Fks2 hot spot substitution, Fks2-S663P, in yeast recovered from all mice (10/10) in that group. The burdens of the 0.5 and 5 mg/kg groups were unchanged (Fig. 1), and *FKS* mutants (Fks2-S663P and Fks2-P667T) were recovered from only one mouse (10%) in the 5 mg/kg treatment group. Of note, the 5 mg/kg dose of caspofungin is considered the equivalent humanized (therapeutic) dose based on previous pharmacodynamics studies (24, 25), and the low echinocandin resistance rate observed following therapeutic dosing is in alignment with clinical resistance rates (3 to 12%) reported from U.S. clinics (5, 6). However, multiple clinics have begun to study the safety and efficacy of high-dose caspofungin (2 to 3 times therapeutic levels) in specific patient subsets (26, 27).

To determine if clinical strains also colonize and respond in a similar fashion to drug exposure, mice were colonized with a range of clinical strains containing differing Msh2 profiles (see Fig. S1 in the supplemental material) and then treated daily with high-dose (20 mg/kg) caspofungin. A 1.5- to 2.5-log decrease in average burden between days 5 and 9 was observed for each group (Fig. S1), and like the Δ*msh2* mutant strain, the burden levels returned to baseline by day 11, as in the previous experiment (Fig. 1). Therefore, clinical strains of C. glabrata colonize and respond to high-dose caspofungin similarly to the Δ*msh2* mutant strain, which was used for subsequent experiments. Further analysis of resistance and breakthrough following colonization with multiple strain backgrounds will be addressed in a future study.

**FIG 2:**
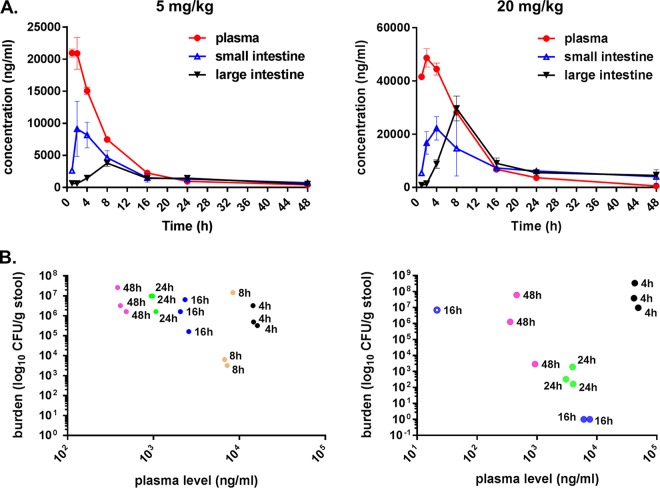
A single dose of 20 mg/kg caspofungin causes greater drug penetration into the GI tract and further reduces GI yeast burdens than that of the therapeutic dose (5 mg/kg). (A) Average caspofungin drug concentrations detected in the plasma, small intestine, and large intestine, following a single dose (i.p.) of 5 mg/kg or 20 mg/kg caspofungin. The mean concentrations from three mice per time point ± standard error of the mean (SEM) are shown. (B) GI yeast burden levels compared to plasma drug levels for each mouse at each time point. In panels A and B, 5 mg/kg and 20 mg/kg data are depicted in the left and right graphs, respectively.

Because of the dose-dependent differences on GI burdens and resistance emergence, we measured the pharmacokinetics of caspofungin within the GI tract following a single-dose administration of 5 or 20 mg/kg. Importantly, the intestinal walls were excluded from the analysis, allowing us to understand the amount of systemically administered drug that successfully traversed into the intestinal lumen. Overall, caspofungin enters the GI tract in a delayed manner and at a reduced level relative to plasma (Fig. 2). For both doses, the maximum concentrations of drug in serum (*C*_max_) of caspofungin were observed at 2, 4, and 8 h postdose for plasma, the small intestine, and the large intestine, respectively (Fig. 2). As expected, treatment with high-dose (20 mg/kg) caspofungin caused greater drug penetration into the GI tract than that with the therapeutic dose (5 mg/kg) (large intestine mean *C*_max_, 36.2 versus 3.8 μg/ml, respectively; small intestine mean *C*_max_, 22.2 versus 9.1 μg/ml, respectively), although the GI drug concentrations were significantly lower than the plasma levels (Fig. 2). In mice treated with the high-dose drug, GI burden levels decreased by 16 h and then returned to original baseline levels by 48 h (Fig. 2). GI burdens in the low-dose injection group exhibited a minimal decrease and faster recovery to baseline (Fig. 2). As expected, GI burden levels were inversely related to the antifungal drug levels within the large intestine (Fig. 2). From these data, we conclude that daily administration of high-dose (20 mg/kg) caspofungin resulted in significantly reduced GI burdens, but drug levels within the GI tract were not maintained at a high level for a sufficiently long time, potentially creating a niche to allow drug adaptation of C. glabrata for regrowth and priming cells for potential resistance acquisition.

### Systemic dissemination from GI colonization upon immunosuppression.

**FIG 3:**
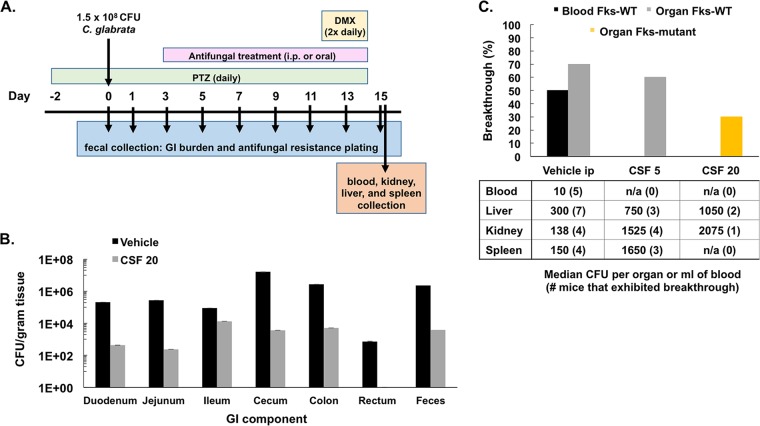
Echinocandin treatment alters breakthrough rates that are successfully achieved following dexamethasone immunosuppression. (A) Model of GI colonization, including immunosuppression. (B) Representative levels of colonization in each GI component with and without 4 days of 20 mg/kg caspofungin (CSF 20) treatment. One mouse per condition was sacrificed, and mean ± standard deviations of 2 or more CFU counts are shown. (B) Breakthrough rates and yeast Fks phenotypes recovered from the blood and organs of mice (10/group) treated (i.p.) with vehicle control (PBS), 5 mg/kg CSF, or 20 mg/kg CSF, as described in panel A. Also shown are the median numbers of CFU identified from the blood and organs of mice that exhibited breakthrough.

In order to recapitulate the natural progression from colonizer to pathogen, we administered an immunosuppressive regimen following antifungal treatment. This treatment simulates the antifungal prophylaxis or empirical therapy that high-risk transplantation patients receive prior to or during immunosuppression (8, 28). Fecal burdens and resistance rates were tracked throughout the experiment as described above and as shown in Fig. 3A. Colonization was established throughout the GI tract but was concentrated within the mouse cecum and colon (Fig. 3B), as previously described for C. albicans colonization (29). Following 4 days of 20 mg/kg caspofungin treatment, colonization levels decreased throughout the GI tract by an average of 2.7 log, and this decrease was mirrored by a 2.8-log decrease in fecal burden (Fig. 3B). Four days of high-dose treatment preceded any observed rebound in fecal burden or emergence of resistance (Fig. 1).

To induce immunosuppression, the corticosteroid dexamethasone (DMX) was administered twice daily from days 12 to 14 prior to euthanasia (day 15; Fig. 3A). Blood, kidneys, liver, and spleen were collected on day 15 and plated to determine CFU levels. Bloodstream infections were identified in 50% of mice (5/10) from the vehicle control group, while no isolates were recovered from the blood of any mouse treated with 5 or 20 mg/kg caspofungin (Fig. 3C). Yeast were recovered from at least one organ in 70%, 60%, and 30% of vehicle, 5 mg/kg CSF-treated, and 20 mg/kg CSF-treated mice, respectively (Fig. 3C). All yeast recovered from the blood and organs of vehicle and 5 mg/kg CSF-treated mice contained wild-type sequences for *FKS1* and *FKS2* hot spots, while yeast recovered from the organs of 20 mg/kg CSF-treated mice exhibited an Fks2 amino acid substitution (Tables 1 and 2). Mutations were determined by melt curve analyses using allele-specific molecular beacons designed to the hot spot regions of *FKS1* and *FKS2* (Fig. S2).

**TABLE 1:** Fks1 and Fks2 hot spot 1 amino acid substitutions identified in yeast recovered from feces, blood, and organs^*a*^

Treatment group	Fks amino acid substitution (no. of mice)						
	Feces					Blood	Organs
	Day 7	Day 9	Day 11	Day 13	Day 15		
CSF 5							WT (2)
CSF 5 + Nz				L630R (3)	L630R (4)		WT (2)
CSF 20		WT (4)	WT (1)	WT (1)			WT + P667T (1)
		P667T (1)	P667T (2)	P667T (4)			P667T (1)
			F659Y (2)	F659Y (4)			
				WT + F659Y (1)			
CSF 20 + Nz	L630R (1)	L630R (4)	L630R (9)	L630R (9)	L630R (9)		L630R (2)
Nz only						WT (1)	WT (1)

a L630 is present in Fks1, and P667 and F659 are present in Fks2. Substitutions identified in prior experiment (e.g., S663P) are described in the text.

**TABLE 2:** Mixed populations recovered from the GI tract of a 20 mg/kg CSF-treated mouse at day 15

Fks amino acid substitution	No. of colonies tested					
	Duodenum	Jejunum	Ileum	Cecum	Colon	Rectum
P667T	2	4	2	3	2	3
WT	2		2	1	2	1

Discontinuation of antifungal treatment during immunosuppression resulted in higher breakthrough rates, including bloodstream infections (Fig. S3); however, we decided to maintain antifungal treatment throughout immunosuppression to better mimic the clinical situation. Immunosuppression with cyclophosphamide failed to result in systemic dissemination (data not shown), as previously described (30). Collectively, the inclusion of a DMX immunosuppressive regimen following colonization results in successful breakthrough of GI colonizers, and treatment with caspofungin decreases these dissemination rates.

### Combination treatment of caspofungin and nikkomycin decreases systemic dissemination but not resistance.

**FIG 4:**
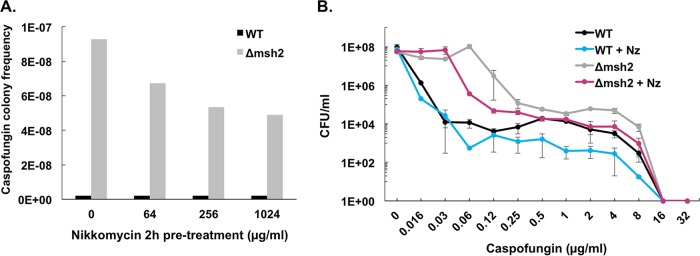
Nikkomycin reduces C. glabrata caspofungin resistance and tolerance formation *in vitro*. (A) Caspofungin-resistant colony frequencies following selection on agar plates containing 2 μg/ml caspofungin. Cells were incubated with the indicated concentrations of nikkomycin Z for 2 h prior to plating. (B) Killing assay with strains grown in the presence or absence of 128 μg/ml nikkomycin Z (Nz) and increasing concentrations of caspofungin. The mean counts from three independent biological experiments ± standard deviations are shown.

**FIG 5:**
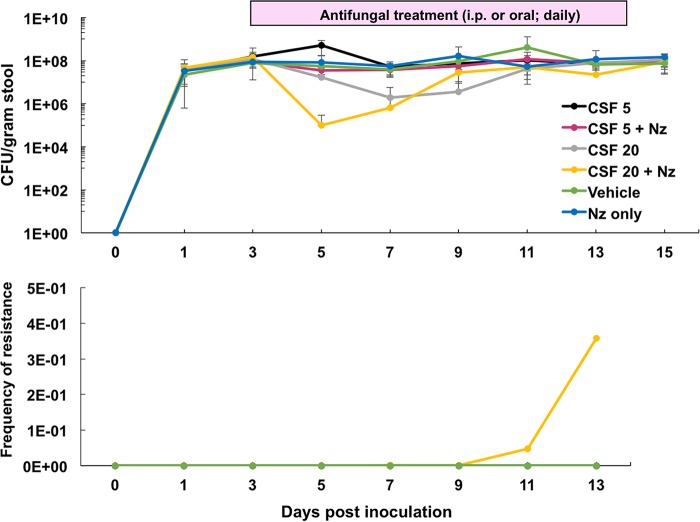
Nikkomycin plus high-dose (20 mg/kg) daily treatments of caspofungin further reduce GI burdens but also lead to *FKS* resistance acquisition. Average GI burdens (top) and average frequencies of resistance (bottom) for each treatment group (10 mice per group, except CSF 20 plus Nz group, which contained 9 mice). See Tables 1 and 2 and Fig. S2 for mutational analysis. Control mice were treated (i.p.) with PBS or nikkomycin (100 mg/kg; oral) alone.

Echinocandin action on yeast cells is well established to induce compensatory responses involving enhanced chitin biosynthesis (17). Nikkomycin Z is a chitin synthase inhibitor that demonstrates *in vitro* synergy when combined with echinocandins toward C. albicans (31), and increased chitin content in C. glabrata leads to incomplete killing by caspofungin (16, 32). Additionally, we found that pretreating our Δ*msh2* mutant cells with nikkomycin reduced the frequency of caspofungin-resistant mutants (Fig. 4A) and increased the killing ability of caspofungin across a range of concentrations for both wild-type and Δ*msh2* cells grown in the presence of nikkomycin (Fig. 4B). Therefore, we utilized our colonization model to assess the effects of nikkomycin on gut burdens, resistance, and dissemination. Mice treated with both nikkomycin (100 mg/kg, oral) and high-dose caspofungin (20 mg/kg, i.p.) exhibited a faster (after 3 days of treatment) and greater (3-log) reduction in GI burden than with high-dose caspofungin treatment alone (Fig. 5, top). However, this combination treatment group also resulted in a burden rebound by day 7 (Fig. 5, top) that correlated with the emergence of resistance (Fig. 5, bottom) and a novel Fks1-L630R amino acid substitution (Table 1 and Fig. S2). To our knowledge, this is a previously undescribed substitution encoded by a T1889G mutation that lies within the hot spot 1 area of *FKS1*. Measurement of glucan synthase inhibition confirmed that this amino acid change confers enzymatic resistance to echinocandins (Fig. S4).

**FIG 6:**
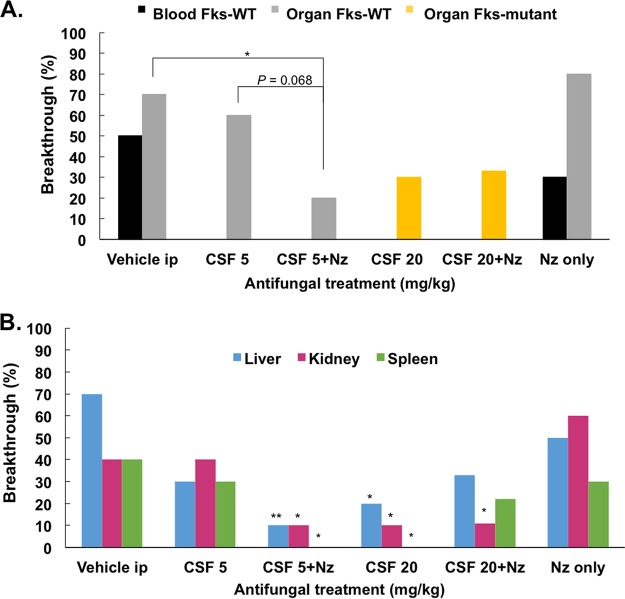
Decreased breakthrough rates observed following antifungal treatment. (A) Percentage of mice from each treatment group that exhibited yeast in blood or organs following immune suppression. *FKS* genotype (WT or mutant) also shown. (B) Percentage of mice exhibiting breakthrough for each organ. Statistics compare indicated organ to same category in the vehicle control group. *, *P* < 0.05; **P < 0.01 (chi-square analysis).

In this experiment, the high-dose caspofungin group (without nikkomycin cotreatment) also demonstrated an initial decrease in burden and subsequent rebound back to baseline colonization (Fig. 5) as observed in the previous experiment (Fig. 1); however, we noticed a lack of colonies on our resistance plates. Molecular beacon analysis demonstrated that *FKS* mutations were present, explaining the burden rebound, but the identified amino acid substitutions (Fks2-P667T and Fks2-F659Y) yielded weaker phenotypes and insufficient growth on the 2 μg/ml caspofungin plates. Interestingly, we found a mixed *FKS* genotype population within mice from this treatment group in the feces, throughout the GI tract, and in organs following immunosuppression (Tables 1 and 2). No bloodstream isolates were identified in any mouse treated with caspofungin (Fig. 6A). Mice that were treated with high-dose caspofungin with or without nikkomycin yielded 33% (3/9 mice) and 30% (3/10 mice) organ dissemination rates, respectively, compared to the 70% observed in the vehicle control group (Fig. 6A), although these yeast contained the same *FKS* mutations that were identified in the fecal matter (Table 1). The nikkomycin plus therapeutic caspofungin (5 mg/kg) treatment produced no change in burden levels (Fig. 5) but significant decreases in systemic breakthrough rates (Fig. 6A and B). Additionally, these breakthroughs contained wild-type *FKS* sequences (Fig. 6A and Table 1). However, the Fks1-L630R substitution was discovered in the feces of mice from this group beginning on day 13 (Table 1), indicating that if immunosuppression began any later, the *FKS* mutants may have disseminated.

These experiments demonstrated that combination treatment with high-dose (20 mg/kg) caspofungin and the chitin synthase inhibitor nikkomycin Z further reduces GI burdens, but the breakthrough and *in vivo* resistance rates were similar to those with high-dose caspofungin treatment alone. Alternatively, treatment with the therapeutic dose of caspofungin (5 mg/kg) plus nikkomycin yielded no change in burden but did significantly reduce breakthrough rates. However, yeast containing a novel *FKS1* mutation arose in mice from both combination treatment groups.

## DISCUSSION

The GI tract is a main reservoir of Candida colonization and has been suggested to be a primary site of resistance development in patients undergoing antifungal prophylaxis and treatment. To address this critical issue, we have established a GI colonization and systemic dissemination model for Candida glabrata that includes measurements of GI burden levels, resistance frequencies, and systemic breakthrough rates. Our studies demonstrate that the gut is a reservoir where robust resistance can develop, and upon immunosuppression, these mutants have the ability to disseminate, or break through, to other parts of the body. The humanized dose (5 mg/kg) of caspofungin yielded little resistance (0 to 10%), although it should be noted that treatment for longer periods of time (10 to 13 days of treatment) may increase the rates of acquired resistance to caspofungin (19). Additionally, these low resistance rates mirror the currently reported echinocandin resistance rates (3 to 12%) reported in the clinic setting (5, 6), supporting the model's clinical significance. To reduce GI burdens and subsequent dissemination, mice were treated with a high dose of echinocandin (20 mg/kg; 4-fold greater than the equivalent humanized dose). An initial burden decline was followed by a rebound to the original colonization levels characterized by a high level of resistant yeast (Fig. 1 and 5). The increased drug pressure led to a more robust adaptation response from the gut population. In a previous C. albicans colonization study (33), treatment of mice with echinocandins at near-humanized doses led to a sustained decrease in GI colonization, indicating an inherent difference between the species in terms of colonization fitness in mice (see reference 34) and response to drug pressure and/or technical differences between models. For example, our model requires a daily antibiotic regimen to maintain high levels (10^7^ to 10^8^ CFU/g of stool) of colonization, while the C. albicans study established a low level (10^4^ CFU/g of stool) of colonization in the absence of antibiotics. Repopulation of the murine GI tract with natural bacterial colonizers following an initial decrease in yeast burdens may affect the ability of the yeast to reestablish colonization in the absence of antibiotics.

One of the major determinants of systemic dissemination of C. glabrata gut colonizers upon immunosuppression is the genetic composition of the yeast population present within the GI tract. We found that the *FKS* genotypes present in the GI tract at the beginning of immunosuppression were also found in the blood and organs, confirming that colonizing strains can become infecting strains. Of note, bloodstream isolates were only isolated from caspofungin-treated mice if we ceased antifungal treatment during immunosuppression (Fig. 6 and S3). We also found that mixed genotypes could arise in the gut (Tables 1 and 2), particularly if a phenotype (i.e., Fks2-P667T) that leads to low-level resistance (<2 μg/ml) develops. This finding is consistent with clinical studies involving serial isolates from patients with recurrent bloodstream infections that show breakthrough isolates with different *FKS* alleles (35, 36). Most of our studies included colonization with the Δ*msh2* mutator strain. Although we have identified that over half of all C. glabrata clinical isolates contain a partial loss-of-function *MSH2* mutation (19), mutational rates of clinical strains are not as high as that of the Δ*msh2* mutant (data not shown). However, the use of the mutator strain aided in our goal to determine if resistance can arise in the gut and if those resistant mutants have the ability to disseminate. Various clinical strains of C. glabrata can effectively colonize the GI tract in this model (Fig. S1), and we plan to test if additional antifungal tolerance mechanisms or other genetic factors (e.g., *PDR1* mutations) influence GI colonization, resistance, and breakthrough.

Another key determinant of systemic breakthrough is plasma and tissue drug levels. Fungal burden levels and resistance rates depended on levels of drug in the GI tract: the lower therapeutic dose (5 mg/kg) of caspofungin had no effect on burden and resulted in low resistance rates, while the high caspofungin dose (20 mg/kg) resulted in a reduction in burden levels but high resistance rates. Insufficient issue penetration of antifungals is a source of concern when treating specific cases of candidiasis (37). Following a single therapeutic dose of the systemically administered caspofungin drug, our pharmacokinetics (PK) studies demonstrated a mean *C*_max_ of 3.8 μg/ml within the large intestine, while a 20 mg/kg dose led to a mean *C*_max_ of 36.2 μg/ml in the same intestinal compartment. The largest decreases in burden corresponded to peak drug levels within the large intestine, indicating a primary reservoir of colonization, as supported by our data (Fig. 3B). The potential gut reservoir of C. glabrata should be taken into consideration during antifungal treatment, particularly in patients with previous azole or echinocandin exposure where C. glabrata may have been preselected over other more susceptible strains, such as C. albicans. A caveat of C. glabrata gastrointestinal colonization mouse models includes the necessary administration of antibiotics (e.g., piperacillin-tazobactam [PTZ]) to attain high levels of colonization (38). Changes in the normal bacterial flora of mice may influence Candida and allow sufficient biofilm formation. However, this may also properly reflect the patient population most at risk for C. glabrata infections. In fact, a study found that treatment with either vancomycin or PTZ antibiotics was a significant risk factor for developing C. glabrata candidemia at a U.S. hospital (39); again, this points to the importance of the Candida reservoir within the GI tract of humans. Overall, the elevated drug treatments led to lower breakthrough rates, likely due to greater drug exposure in these tissues; however, because resistance had already developed in the gut, yeast that were recovered from organs of high-dose treatment groups contained the same *FKS* mutations (Tables 1 and 2).

Multiple factors, including penetration of drug, anatomical complexity of the GI tract, and the capacity of C. glabrata to survive and adapt to these drug concentrations *in vivo*, likely contributed to the inability of caspofungin to completely decolonize or sterilize the gut. To this end, we attempted to use a combinatorial treatment that showed promise against C. glabrata persistence and mutant formation *in vitro* (Fig. 4). As expected, treatment with the chitin synthase inhibitor nikkomycin Z resulted in an even greater reduction of GI burden when combined with high-dose daily caspofungin (20 mg/kg); however, resistance (*FKS* mutations) arose quickly (within 5 days of treatment) (Fig. 5 and Table 1). Nikkomycin treatment combined with the therapeutic dose (5 mg/kg) of caspofungin did not affect burden levels but did significantly decrease organ breakthrough rates (Fig. 6). Additionally, yeast that were recovered from organs of mice in this group were *FKS* wild type (Table 1). However, caution must be taken in the interpretation of these results, since we did identify *fks1* mutant alleles from the feces of mice in this group, albeit late in the experiment (day 13) after immunosuppression was started (Table 1). Interestingly, a novel Fks1 substitution (L630R) was isolated from mice in the nikkomycin plus caspofungin treatment groups and may reflect the dual pressure of these drugs. More studies must be done to determine the *in vivo* effects of combination treatment of C. glabrata infection and colonization. The marked ability of C. glabrata populations to tolerate or adapt to various antifungals, and other compounds should be considered when attempting to develop treatments that target tolerance pathways within this organism.

The experiments presented here were performed with caspofungin, and although we plan to determine the effects upon treatment with other echinocandins, including micafungin and anidulafungin, it is worth noting that the *FKS* mutations identified within our mouse model have also been identified within patient isolates following treatment failure with any echinocandin (5, 40). In summary, we have demonstrated that echinocandin treatment can lead to the development of resistant mutants (*fks1*/*fks2*) within the GI tract and that those mutants can breakthrough systemically upon immune suppression within a mouse model of C. glabrata GI colonization and dissemination.

## MATERIALS AND METHODS

### Ethics statement.

Mice were housed in the Public Health Research Institute's Animal Biosafety Level-2 Research Animal Facility (ICPH RAF), a center of the New Jersey Medical School, Rutgers University (NJMS-Rutgers). The animal facility follows the Public Health Service and National Institute of Health Policy of Humane Care and Use of Laboratory Animals guide. All experimental protocols were approved by the Rutgers Institutional Animal Care and Use Committee (IACUC).

### Gastrointestinal C. glabrata colonization mouse model.

Six-week-old female outbred CF-1 immunocompetent mice (Charles River Laboratories) were treated (subcutaneously [s.c.]) with 320 mg/kg of piperacillin-tazobactam (PTZ; 8:1 ratio) beginning on day −2 to clear native intestinal bacterial flora. Daily PTZ treatment was maintained throughout the experiment (see Fig. 3A). On day 0, mice were inoculated via oral gavage with approximately 1.5 × 10^8^ CFU of C. glabrata in 100 μl of saline. Mice were colonized with ATCC 2001 (CBS 138), clinical strains from the Perlin Laboratory collection, or mutator strain 2001 Δ*msh2 (*19). Fresh fecal samples were collected throughout the experiment to assess fungal burden in the GI tract. Daily administration of caspofungin (0.5, 5, or 20 mg/kg, i.p.), nikkomycin Z (100 mg/kg, oral) (Sigma), or saline (100 μl i.p.) was initiated on day 3 postinoculation and continued through sacrifice day (typically day 15). Caspofungin-resistant colony frequencies were determined through selection of fecal samples on yeast extract-peptone-dextrose (YPD) plates supplemented with caspofungin (2 μg/ml), PTZ (16 μg/ml), and chloramphenicol (20 μg/ml). The corticosteroid dexamethasone (DMX) was administered twice daily (100 mg/kg, i.p.) for 3 days (days 12 to 14) prior to euthanization (day 15; Fig. 3A). Blood, kidneys, liver, spleen, and the gastrointestinal tract components were collected on day 15 (or earlier where indicated), homogenized, and plated onto YPD plates supplemented with chloramphenicol (75 μg/ml) and ampicillin (50 μg/ml) to determine CFU levels.

### Rapid detection of *FKS* mutations.

Colonies obtained from feces, blood, and organs were screened for *FKS*-associated echinocandin resistance by allele-discriminating real-time PCR, as described in reference 41. Briefly, two sets of asymmetric PCR primers were used to amplify the hot spot 1 (HS1) regions of *FKS1* and *FKS2* via colony PCR. Two molecular beacon probes were designed to complement the wild-type (WT) (ATCC 2001) genotype in the target region (*FKS1* HS1 or *FKS2* HS1) but possess various binding energies to non-WT sequences. Immediately after amplification, melting curve analysis was performed with a Mic quantitative PCR (qPCR) cycler (Bioline) at 95°C for 3 min and then 40°C for 30 s, after which it was melted from 50°C to 70°C with a ramp rate of 0.025°C/s. Due to the stability difference of the probe-target hybrids, characteristic profiles are produced for different *FKS* genotypes in the subsequent melting curve analysis (see Fig. S2). Any colonies that generated questionable melt curves were subjected to traditional PCR amplification and sequencing to confirm *FKS* genotype.

### Pharmacokinetics.

Mice were colonized with ATCC 2001 and then treated (i.p.) once with 5 or 20 mg/kg caspofungin at time zero (2 days postinoculation). Three mice from each treatment group were sacrificed at each time point (0, 1, 2, 4, 8, 16, 24, and 48 h), and blood and small and large intestinal lumen contents (without intestinal walls) were collected. Fecal pellets were also collected from mice at 0, 4, 16, 24, and 48 h prior to sacrifice. Caspofungin levels in plasma and GI compartments were measured by liquid chromatography-tandem mass spectrometry (LC-MS/MS) in electrospray positive-ionization mode (ESI+) on an AB Sciex QTrap 4000 triple-quadrupole mass spectrometer combined with an Agilent 1260 high-performance liquid chromatograph (HPLC) using the Analyst software and multiple-reaction monitoring (MRM) of precursor/product transitions. The MRM transitions used were 547.50/538.40 for caspofungin and 455.4/165.2 for the internal standard verapamil. Chromatography was performed with an Agilent Zorbax SB-C_8_ column (2.1 by 30 mm; particle size, 3.5 μm) using a reverse-phase gradient elution. One-tenth percent formic acid in Milli-Q deionized water was used for the aqueous mobile phase and 0.1% formic acid in acetonitrile (ACN) for the organic mobile phase. Small and large intestinal contents were homogenized prior to extraction by combining 3 parts PBS buffer to 1 part GI tract contents. One milligram per milliliter dimethyl sulfoxide (DMSO) stock was serially diluted in 50/50 ACN-water to create standard curves and quality control spiking solutions. Twenty microliters of neat spiking solutions was added to 20 μl of drug-free mouse K_2_EDTA plasma (Bioreclamation) or GI tract homogenate, and extraction was performed by adding 200 μl of acetonitrile-methanol 50/50 protein precipitation solvent containing 10 ng/ml verapamil (Sigma). Extracts were vortexed for 5 min and centrifuged at 4,000 rpm for 5 min. The supernatants were analyzed by LC-MS. Sample analysis was accepted if the concentrations of the quality control samples were within 20% of the nominal concentration.

### *In vitro* killing assay.

Fresh 1-ml RPMI cultures (plus necessary amino acids) of C. glabrata (1 × 10^7^ cells) were incubated at 37°C while shaking (165 rpm) for 24 h in 2-fold increasing concentrations (0.016 to 32 μg/ml) of caspofungin and in the presence (128 μg/ml) or absence of nikkomycin Z. After 24 h, 100 μl of the appropriate dilutions for each culture was plated onto YPD plates. CFU were counted 24 h after plating and data represented as the CFU per milliliter that survived at each concentration of drug.

### Glucan synthase assay.

Candida glabrata 2001 Δ*msh2* and 2001 Δ*msh2* Fks1-L630R (a novel mutant recovered from colonized mice) were grown with vigorous shaking at 37°C to early stationary phase in YPD (1% yeast extract, 2% peptone, 2% dextrose) broth, and cells were collected by centrifugation. Cell disruption, membrane protein extraction, and partial 1,3-β-d-glucan synthase purification by-product entrapment were performed as previously described (42). Reactions were initiated by the addition of product-entrapped glucan synthase. Sensitivity to caspofungin and micafungin was measured in a polymerization assay using a 96-well 0.65-μm multiscreen HTS filtration system (Millipore Corporation, Bedford, MA) in a final volume of 100 μl, as previously described (43). Serial dilutions of the drugs (0.01 to 10 000 ng/ml) were used as calibration standards. Antifungals were dissolved in water. Inhibition profiles and 50% inhibitory concentration (IC_50_) values were determined using a normalized response (variable-slope) curve-fitting algorithm with the GraphPad Prism software.

### Statistics.

All data analyses were performed using GraphPad Prism, version 6.05, software for Windows (GraphPad Software, San Diego, CA). Chi-square analysis (χ^2^) was used to determine breakthrough rate differences between treatment groups. A *P* value of <0.05 (two-tailed) is considered statistically significant.

## Supplementary Material

Supplemental material
