# Supplementary material for: The Gastrointestinal Tract Is a Major Source of Echinocandin Drug Resistance in a Murine Model of Candida glabrata Colonization and Systemic Dissemination
Source: Antimicrob Agents Chemother. 2017 Nov 22;61(12):e01412-17. doi: 10.1128/AAC.01412-17 (PMC5700336; doi:10.1128/AAC.01412-17)
Supplement: Supplemental material [file supp_61_12_e01412-17__index.html]

Supplemental material 

# The Gastrointestinal Tract Is a Major Source of Echinocandin Drug Resistance in a Murine Model of Candida glabrata Colonization and Systemic Dissemination

## Supplemental material

- Supplemental file 1 -

  Supplemental Figures S1 to S4

  PDF, 532K
