# Supplementary material for: The Gastrointestinal Tract Is a Major Source of Echinocandin Drug Resistance in a Murine Model of Candida glabrata Colonization and Systemic Dissemination
Source: Antimicrob Agents Chemother. 2017 Nov 22;61(12):e01412-17. doi: 10.1128/AAC.01412-17 (PMC5700336; doi:10.1128/AAC.01412-17)
Supplement: Supplemental material [file AAC.01412-17_zac012176718s1.pdf]

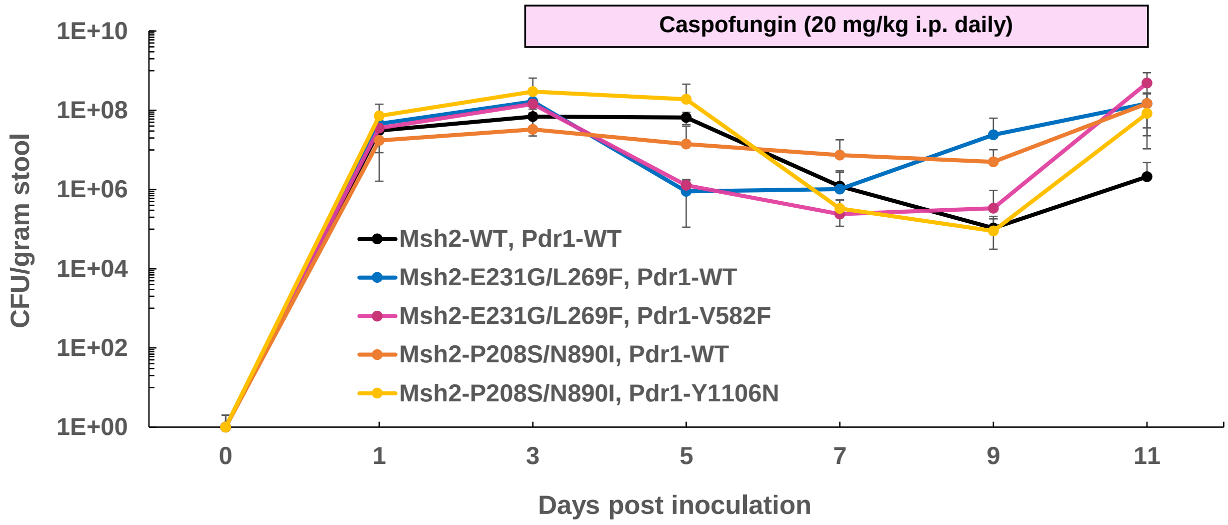

Supplementary figure 1. GI colonization with clinical isolates of *C. glabrata*. Isolates with differing Msh2 and Pdr1 profiles colonized mice (5 mice per group) to similar levels as in the previous experiments. A reduction in burden was observed following high dose caspofungin treatment (20 mg/kg; i.p.) for all groups between days 5 and 9 with rebounds by day 11 for all strains. Amino acid differences compared to ATCC2001/CBS138 are shown in the legend.

A.

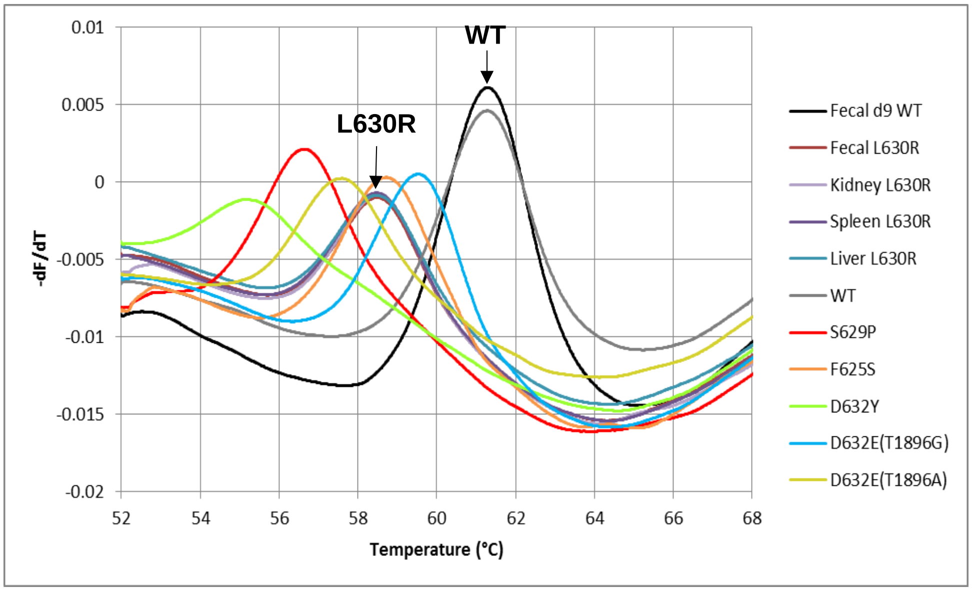

Sample Name	Tm (°C)
Fecal d9 WT	61.29
WT	61.28
D632E(T1896G)	59.53
F625S	58.71
Kidney L630R	58.5
Fecal L630R	58.49
Spleen L630R	58.47
Liver L630R	58.47
D632E(T1896A)	57.57
S629P	56.64
D632Y	55.19

B.

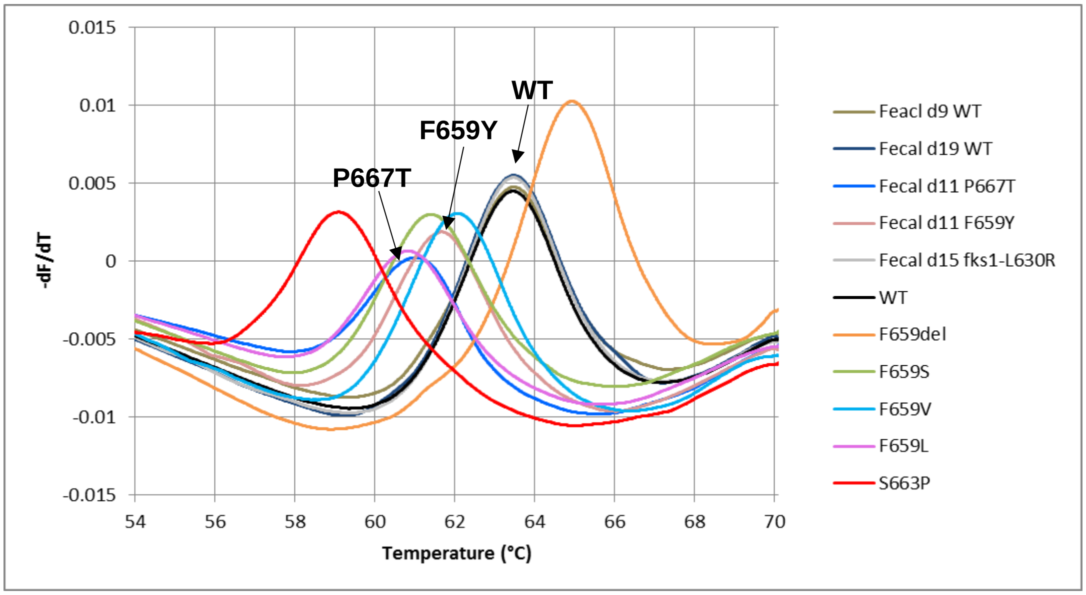

Sample Name	Tm (°C)
F659del	64.94
Feacl d9 WT	63.48
Fecal d19 WT	63.48
Fecal d15 fks1-L630R	63.47
WT	63.46
F659V	62.07
Fecal d11 F659Y	61.66
F659S	61.41
Fecal d11 P667T	61.01
F659L	60.83
S663P	59.09

Supplementary figure 2. *FKS* melt curve analyses using allele-specific molecular beacons. *FKS1* (**A**) and *FKS2* (**B**) melting curves and temperatures of yeast recovered from feces and organs of colonized mice. See methods for additional information.

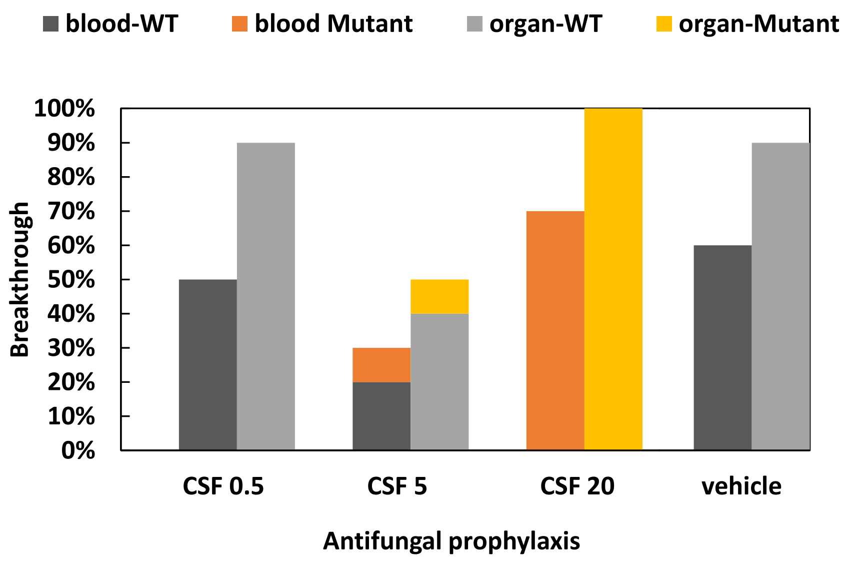

Supplementary figure 3. Removal of antifungal treatment during immunosuppression yields elevated breakthrough rates. Caspofungin (CSF) treatment (0.5, 5, or 20 mg/kg) was stopped during the three days of dexamethasone treatment prior to sacrifice. Bar colors represents the genotype (wild type or mutant *FKS*) of recovered yeast.

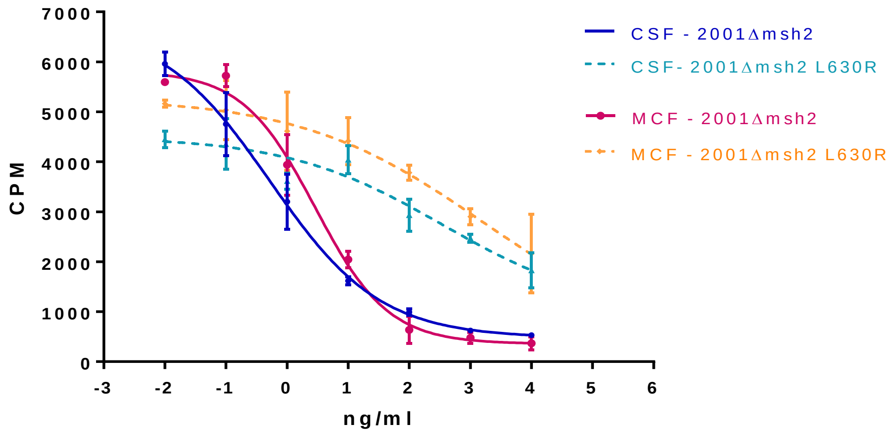

	CSF - 2001Δmsh2	CSF- 2001Δmsh2 L630R	MCF - 2001Δmsh2	MCF - 2001Δmsh2 L630R
EC50	0.5245	351.4	2.878	1510

Supplementary figure 4. Glucan synthase containing Fks1-L630R yields enzymatic resistance. Half maximal inhibitory concentration (IC_{50}) values for susceptible (2001Δmsh2) and resistant (L630R) *C. glabrata* strains. IC_{50} values shown below the graph. CPM: counts per minute; CSF: caspofungin; MCF: micafungin.
